# Cooperative Anti-Invasive Effect of Cdc42/Rac1 Activation and ROCK Inhibition in SW620 Colorectal Cancer Cells with Elevated Blebbing Activity

**DOI:** 10.1371/journal.pone.0048344

**Published:** 2012-11-07

**Authors:** Marion de Toledo, Christelle Anguille, Laureline Roger, Pierre Roux, Gilles Gadea

**Affiliations:** 1 Institut de Génétique Moléculaire de Montpellier, Centre national de la recherche scientifique UMR 5535, Montpellier, France; 2 Centre de Recherche en Biochimie Macromoléculaire, Centre national de la recherche scientifique UMR 5237, Montpellier, France; 3 Institute of Cancer and Genetics, Cardiff University School of Medicine, Cardiff, United Kingdom; Southern Illinois University School of Medicine, United States of America

## Abstract

Rho GTPases are key regulators of tumour cell invasion and therefore constitute attractive targets for the design of anticancer agents. Several strategies have been developed to modulate their increased activities during cancer progression. Interestingly, none of these approaches took into account the existence of the well-known antagonistic relationship between RhoA and Rac1. In this study, we first compared the invasiveness of a collection of colorectal cancer cell lines with their RhoA, Rac1 and Cdc42 activities. A marked decrease of active Cdc42 and Rac1 correlated with the high invasive potential of the cell lines established from metastatic sites of colorectal adenocarcinoma (LoVo, SKCo1, SW620 and CoLo205). Conversely, no correlation between RhoA activity and invasiveness was detected, whereas the activity of its kinase effector ROCK was higher in cancer cell lines with a more invasive phenotype. In addition, invasiveness in these colon cancer cell lines was correlated with a typical round and blebbing morphology. We then tested whether treatment with PDGF to restore Cdc42 and Rac1 activities and/or with Y27632, a chemical inhibitor of ROCK, could decrease the invasiveness of SW620 cells. The association of both treatments substantially decreased the invasive potential of SW620 cells and this effect was accompanied by loss of membrane blebbing, restoration of a more elongated cell morphology and re-establishment of E-cadherin-dependent adherens junctions. This study paves the road to the development of therapeutic strategies in which different Rho GTPase modulators are combined to modulate the cross-talk between Rho GTPases and their specific input in metastatic progression.

## Introduction

Rho GTPases are essential for many cell functions, including membrane trafficking, transcriptional activation, apoptosis, cell cycle progression, cell polarity, adhesion and migration [1], [2], [3], [4]. Indeed their deregulation has important consequences in many physio-pathological processes [5]. In particular, Rho GTPases are crucial regulators of cancer progression through modulation of cell proliferation, apoptosis, invasion and metastasis formation [6].

The role of three Rho GTPases in bi-dimensional migration has been well characterized. Rac1 drives motility by promoting lamellipodium formation and cell protrusions [7]. RhoA signalling activates the ROCK family of kinases, promoting formation of Actin stress fibres and generation of the Actomyosin contractile force that is required for retraction of the cell rear in mesenchymal-type movement [8], [9]. Cdc42 is activated at the leading edge of lamellipodia and is required for Arp2/3-dependent Actin assembly [10]. Furthermore, the existence of an antagonistic relationship between Rac1 and RhoA explains the polarized movement during directed cell migration. Interestingly, these two proteins suppress each other activities and phenotypes [11], [12], [13]. Specifically, RhoA and Rac1 activities are spatially and temporally controlled to promote dynamic cytoskeletal changes during migration. Rac1 activity is limited to the leading edge to extend protrusions at the front, whereas RhoA drives contraction at the rear of the migrating cell. RhoA and Rac1 localized effects are presumably driven by specific regulatory mechanisms. Indeed, Wildenberg and colleagues demonstrated that Rac1-dependent down-regulation of RhoA activity is controlled by adherens junction integrity [14].

However, the mechanisms that mediate tumour cell motility are different in more complex three-dimensional matrices. In this context, some tumour cells adopt an amoeboid form of movement [15], [16] that is characterized by a rounded, blebbing cell morphology, independence from extracellular proteases and the requirement of high levels of Actomyosin contractility downstream of the RhoA-ROCK pathway to deform the extracellular matrix and drive cell movement [17], [18], [19], [20]. Studies using intravital microscopy have shown that the amoeboid movement of tumour cells can be very rapid in vivo (∼5 µm/min [21]). Conversely, mesenchymal-type movement is characterized by an elongated morphology, resulting from Rac1-dependent Actin assembly at the leading edge. These two modes of movement are inter-convertible and tumour cells may undergo amoeboid-mesenchymal and mesenchymal-amoeboid transitions [15], [18], [20] that seem to be controlled by an antagonism between the Rac1 and RhoA signalling pathways. The discovery of ROCK-regulated Rac1 GTPase-activating proteins (GAPs) helped understanding how the RhoA/ROCK pathway can suppress Rac1-mediated Actin polymerization [14], [22], [23]. In any case, this plasticity may allow cells to adapt their mode of migration to the different environments and therefore it may be beneficial to tumour cells.

Besides the tight spatio-temporal regulation of the different Rho GTPases, also the activity of their upstream and downstream partners is strictly controlled. Indeed, Rho GTPase effects on cell behaviour can be modulated also via specific interactions with regulators (e.g., Guanine nucleotide exchange factors, GEFs) to regulate apparently incompatible processes. For instance, DOCK10, a Cdc42 GEF, is a key player in amoeboid migration through a DOCK10-Cdc42-Pak2 signalling pathway. Accordingly, expression of activated Cdc42 induces a mesenchymal-amoeboid transition. However, inhibition of Cdc42 results in loss of the mesenchymal morphology, suggesting that Cdc42 plays a role also in mesenchymal-type morphology through different pathways that might implicate other GEFs [24]. In addition, in two-dimensional culture models, RhoA is activated not only at the contractile tail, but also at the leading edge. Indeed, RhoA activity remains high at membrane ruffles in nascent lamellipodia [25]. These observations suggest that RhoA is implicated in the regulation of Rac1-dependent membrane ruffling. Indeed, RhoA cooperates with Rac1 to induce membrane ruffles via the recruitment of its specific effector mDia. Taken together, these data clearly indicate that the cross-talk between these Rho GTPases excludes the hypothesis that each of them is responsible for only one mode of migration.

Rho GTPases regulate also Actin cytoskeletal organization and cell adhesion. E-cadherin, a single-span trans-membrane glycoprotein, establishes homophilic interactions with adjacent E-cadherin molecules that are expressed by neighbouring cells, thereby forming the core of the epithelial adherens junctions [26], [27]. Through its cytoplasmic domain, E-cadherin associates with a number of proteins, including three Catenins (alpha, beta, and p120), which link E-cadherin to the Actin cytoskeleton. The E-cadherin-Catenin complex and the underlying Actin cytoskeleton undergo a series of reorganizations that are controlled by the Rho GTPases Rac1 and RhoA and that result in the expansion and completion of cell-cell adhesion. In view of its role in maintaining adherens junctions and its narrow link with Rho GTPases, E-cadherin loss might promote metastasis by enabling the first step of the metastatic cascade: the loss of cell-cell contacts. Indeed, decreased E-cadherin expression has been associated with tumour invasiveness, metastatic dissemination and poor clinical prognosis [28], [29], [30].

Therefore, Rho GTPases appear to be appealing targets for cancer therapy. Several strategies have been developed to counteract Rho GTPase signalling deregulation during tumorigenesis, such as direct targeting of the Rho GTPase activities [31], [32], or inhibition of downstream effectors, like ROCK, with promising results in the prevention of invasiveness *in vivo* [33]. An alternative strategy consists in targeting regulators of Rho GTPase active state, such as GEFs, GAPs and guanine nucleotide dissociation inhibitors (GDIs) [32] [34]. Although Rho GTPase inhibitors have not yet been widely adopted for clinical use, their potential value as anticancer drugs still drives considerable pharmaceutical research and development [35]. However, the cross-talk between Rho GTPases and their functional duality, which is a key feature of their regulation, have never been considered by these targeting strategies.

In this study, we first compared the invasiveness of a collection of colorectal cancer cell lines with their RhoA, Rac1 and Cdc42 activities. We report that the high invasive potential of colorectal cancer cells with elevated blebbing activity correlates with both increased ROCK activation and decreased Cdc42 and Rac1 activities. Combined treatment with PDGF to restore Cdc42 and Rac1 function and with Y27632 to inhibit ROCK synergistically reduced the invasive potential of such colorectal cancer cells. This provides new insights for the development of anticancer agents that may combine simultaneous inhibition of ROCK and re-activation of Cdc42 and Rac1 to act on both specific and common pathways.

## Results

### The level of active Cdc42 and Rac1, but not of RhoA, is decreased in highly invasive colorectal cell lines

**Figure 1:**
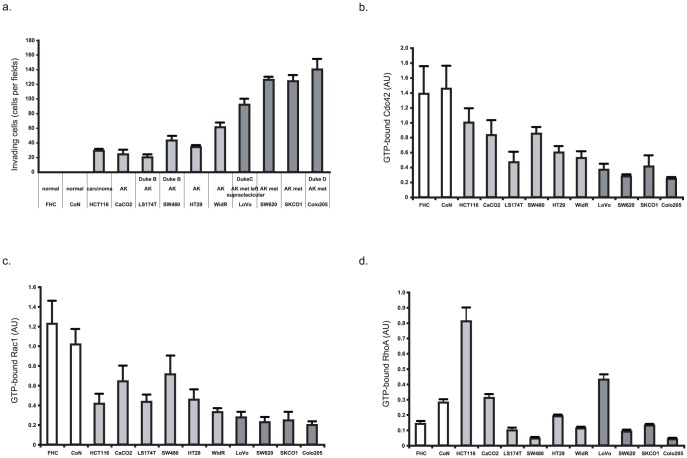
Colon cancer cell invasion correlates with decreased levels of GTP-bound Cdc42 and Rac1. (a) Results of the Matrigel invasion assay for the different colon cancer cell lines. Cells that had invaded through Matrigel were detected on the lower side of the filter by fluorescence and counted as described in Materials and Methods. The values are the mean +/− SD (error bars) of at least three independent experiments. (b) Cells were lysed and the level of GTP-bound Cdc42 was measured as described in Materials and Methods. Cdc42-GTP precipitated with GST-PAK1 and total Cdc42 in the lysates were detected by immunoblotting with an anti-Cdc42 antibody. Values are the mean +/− SD (error bars) of at least three independent experiments. (c) Cells were lysed and the level of GTP-bound Rac1 was measured as described in Materials and Methods. Rac1-GTP precipitated with GST-PAK1 and total Rac1 in the lysates were detected by immunoblotting with an anti-Rac1 antibody. Values are the mean +/− SD (error bars) of at least three independent experiments. (d) Cells were lysed and the level of GTP-bound RhoA was measured as described in Materials and Methods. RhoA-GTP precipitated with GST-RBD and total RhoA in the lysates were determined by immunoblotting with an anti-RhoA antibody. Values are the mean +/− SD (error bars) of at least three independent experiments.

Transition to a highly motile phenotype is a characteristic of invasive cancer cells and an indication that a tumour might metastasize. To classify their invasive potential, we compared the ability of a collection of colorectal cancer cell lines to invade Matrigel, a tri-dimensional matrix that resembles the basement membrane and mimics the complex extracellular environment found in many tissues. We thus assessed the invasiveness of two normal colon cell lines (FHC and CoN), of the HCT-116 colon carcinoma cell line, of the CaCO2, LS174T, SW480, HT29 and WidR cell lines that were derived from colorectal adenocarcinomas and of four cell lines (LoVo, SKCo1, SW620 and CoLo205) established from metastatic sites of colorectal adenocarcinoma. The four cell lines issued from metastatic sites exhibited the highest ability to invade Matrigel, indicating that these cells have retained their ability to invade even after they reached their secondary site (Figure 1a).

We then assessed whether invasiveness and the level of active Rho GTPases, which are key components of the invasive machinery, were correlated by comparing the level of GTP-bound Cdc42, Rac1 and RhoA in the different colorectal cancer cell lines. A marked decrease in active Cdc42 and Rac1 was observed in the most invasive colorectal cancer cell lines (Figure 1b and 1c). Conversely, the level of GTP-bound RhoA was not correlated with cell invasiveness. In particular, a strong RhoA activity was observed in HCT-116 cells, which were characterized by low capability to invade Matrigel (Figure 1d). The variations in the level of active GTPases in the different cell lines were not due to a difference in their protein expression as this was almost equivalent in all cell lines (not shown). We conclude that, in the tested colorectal cancer cell lines, the level of active Cdc42 and Rac1, but not of RhoA, is inversely correlated with the invasiveness of such cells (respective *p values*: 0.0008, 0.0003 and 0.134).

### Cofilin phosphorylation increases with the invasiveness of the different colorectal cancer cell lines

**Figure 2:**
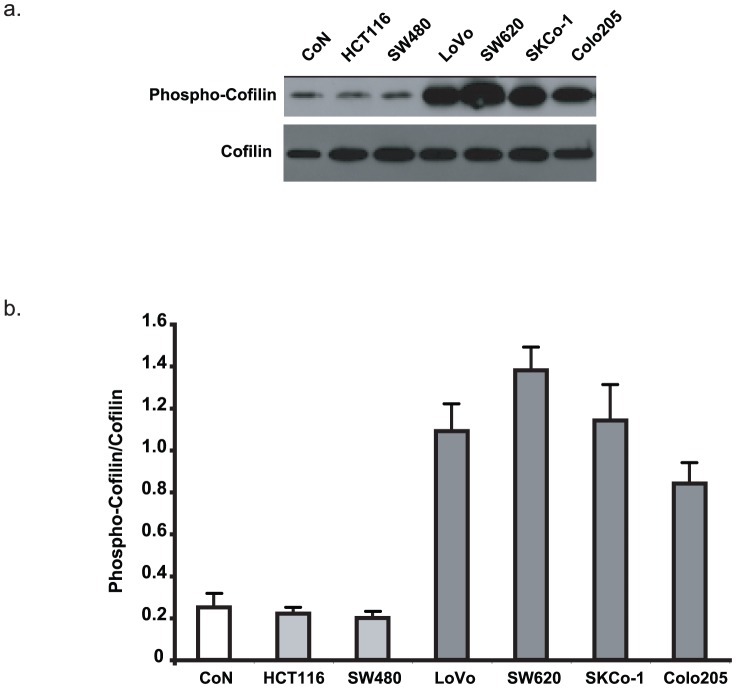
Cofilin phosphorylation increases in the most invasive colon cancer cell lines. Cells were lysed and the amount of phosphorylated Cofilin and of total Cofilin present in the lysates was analysed by immunoblotting. (a) Representative immunoblot. (b) Quantification of Cofilin phosphorylation. Histograms represent the ratios of the phospho-Cofilin signal over the total Cofilin signal. Values are the mean +/− SD (error bars) of three independent experiments.

The finding that RhoA activity was not related with the invasiveness of the different colon cancer cell lines is not in agreement with the frequent involvement of the RhoA pathway in tumour cell invasion, particularly through the activation of its effector kinase ROCK. We thus tested the hypothesis that, in these cell lines, the level of ROCK activation might not mirror the level of GTP-bound RhoA. To this aim, we evaluated ROCK activation in the studied colorectal cancer cell lines by quantifying the phosphorylation status of the ROCK substrate Cofilin, which controls the formation of oriented Actomyosin II bundles in the cell body [36]. Phosphorylated Cofilin was detected by immunoblotting with specific anti-phosphorylated Cofilin antibodies and its expression was normalized to that of total Cofilin (Figure 2a). Cofilin was weakly phosphorylated in the normal colon cell line CoN as well as in the poorly invasive HCT-116 and SW480 cell lines in comparison to the most highly invasive cell lines LoVo, SW620, CoLo205 and SKCo-1, in which phosphorylated Cofilin was very abundant. These results suggest that the level of active ROCK, but not of active RhoA, is associated with colon cancer cell invasiveness.

### Combined restoration of Cdc42 and Rac1 activities and inhibition of ROCK decrease cell blebbing and induce cell elongation

**Figure 3:**
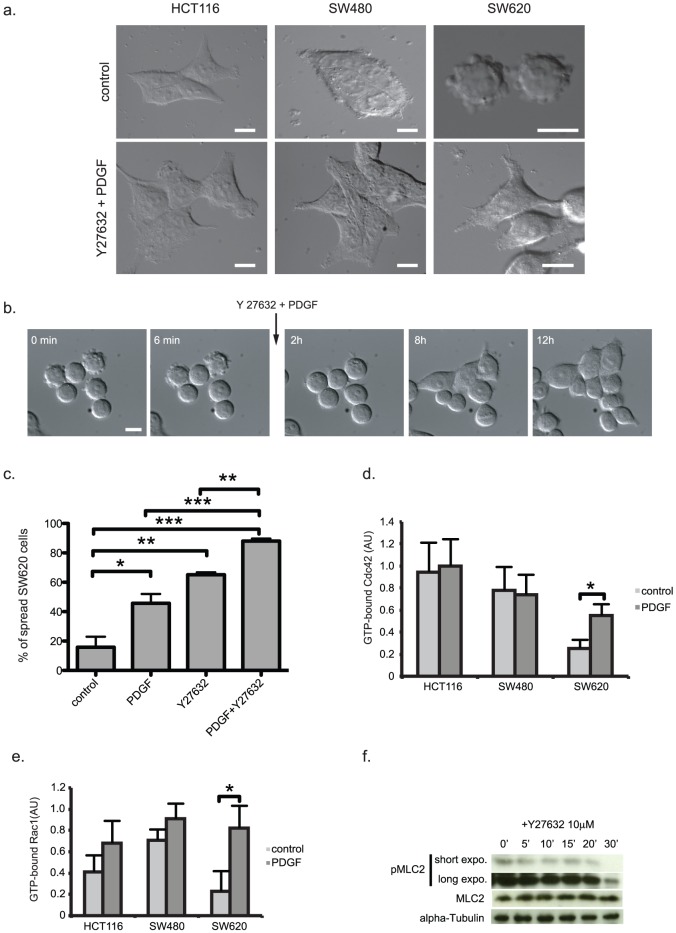
PDGF and Y27632 treatments promote elongation of colon cancer cells. (a) Still DIC time-lapse images of cells treated or not (control) with PDGF and Y27632 (from videos S1, S2, S3, S4, S5, and S6). Frames show the change in morphology (from round to a more elongated shape) of the cells at the beginning of the movies. Scale bars, 10 µm. (b) DIC time-lapse images of SW620 cells before and after treatment with PDGF + Y27632 (from video S7). Frames show the change in cell morphology at the indicated times. Scale bars, 10 µm. (b) Quantification of cell elongation. A cell was considered elongated when its longest dimension was twice the shortest one and when it showed at least one protrusion [23]. Histograms represent the percentage of elongated SW620 cells following treatment or not (control) with PDGF and/or Y27632 for 24 hours. Values are the mean +/− SD (error bars) of three independent experiments and the statistical significance was calculated using the unpaired t-test. * p<0.05, ** p<0.01, *** p<0.001. (d) Cdc42 activity was determined following treatment or not (control) with PDGF for 24 hours as described in Materials and Methods. Values are the mean +/− SD (error bars) of four independent experiments and the statistical significance was calculated using the unpaired t-test. *p<0.05. (e) Rac1 activity was determined following treatment or not (control) with PDGF for 24 hours as described in Materials and Methods. Values are the mean +/− SD (error bars) of four independent experiments and the statistical significance was calculated using the unpaired t-test. *p<0.05. (f) SW620 cells were lysed and the amount of phosphorylated Myosin Light Chain 2 (MLC2) and of total MLC2 present in the lysates was determined by immunoblotting at different time-points (0 to 30 minutes) following treatment with 10 µM Y27632. Shown is a representative immunoblot.

ROCK-dependent Actomyosin contractility is critical for cell morphology. High ROCK activity can also induce dynamic membrane blebbing. To check whether ROCK activation in the different colorectal cell lines was correlated with cell morphology, we monitored cells using differential interference contrast time-lapse microscopy. Whereas HCT-116 and SW480 cells retained some epithelial features (elongated shape, cell-cell contacts), SW620 cells were spherical and exhibited intense peripheral blebbing activity (Figure 3a, upper panels: still photographs from videos S1, S2, S3, S4, S5, and S6), in agreement with elevated Actomyosin contractility due to their much higher ROCK activity in comparison to the other two cell lines (see Figure 2b). To test whether the observed decrease in Cdc42 and Rac1 and increase in ROCK activity were both involved in tumour progression to more advanced stages, we treated these three colon cancer cell lines with PDGF to re-activate Cdc42 and Rac1 and/or with Y27632 to block ROCK. Combined PDGF and Y27632 treatment resulted in the acquisition of a more epithelial phenotype with elongated cells, particularly in the SW620 cell line (Figure 3a, lower panels). Indeed, these cells, which were round and with elevated membrane blebbing at the beginning of the combined treatment, stopped blebbing and flattened out (Figure 3b and video S7). Quantification of the number of elongated cells following the different treatments clearly demonstrated that the combined restoration of Cdc42 and Rac1 activities and inhibition of ROCK had an additive effect (Figure 3c). To monitor PDGF-dependent activation of Cdc42 and Rac1 in the three cell lines, we measured the level of GTP-bound Cdc42 and Rac1 (Figure 3d and e). PDGF did not strongly affect the amount of GTP-bound Cdc42 and Rac1 in HCT-116 and SW480 cells, whereas it clearly increased it in SW620 cells. In addition, we also checked Y27632 effect on Myosin Light Chain 2 (MLC2) phosphorylation, as a readout of ROCK-dependent contractility and found that phosphorylated MLC2 was decreased in Y27632-treated SW620 cells (Figure 3f). Taken together these data suggest that combined treatment with PDGF and Y27632 to restore Cdc42 and Rac1 activity and inhibit ROCK can convert invasive, blebbing SW620 cells to a more epithelial phenotype.

### Combined restoration of Cdc42 and Rac1 activity and inhibition of ROCK re-localize E-cadherin to the cell junctions

**Figure 4:**
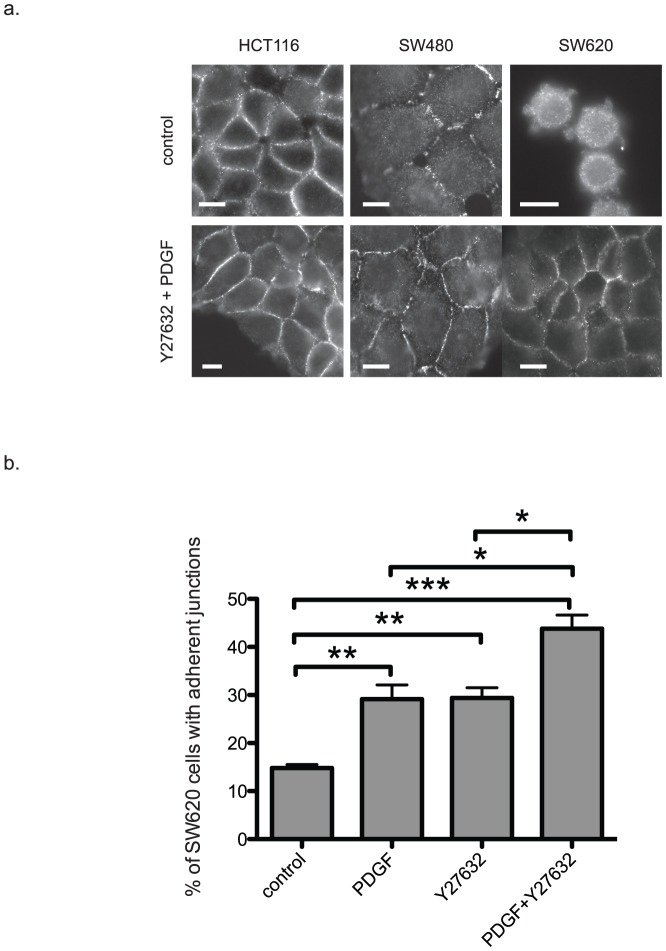
The combined treatment with PDGF and Y27632 induces the re-establishment of E-cadherin junctions. (a) Representative E-cadherin staining in colon cancer cells treated or not (control) with PDGF and Y27632 for 24 hours. Scale bars, 10 µm. (b) Quantification of E-cadherin junctions. We scored as positive every cell showing at least one E-cadherin junction with one or more neighbouring cells. Histograms represent the percentage of SW620 colon cancer cells with E-cadherin junctions following treatment or not with PDGF and/or Y27632 for 24 hours. Values are the mean +/− SD (error bars) of three independent experiments and the statistical significance was calculated using the unpaired t-test. * p<0.05, ** p<0.01, *** p<0.001.

Upon combined treatment with PDGF and Y27632, SW620 cells appeared to have restored adherens junctions (Figure 3a). In order to clarify this point, we assessed the expression of E-cadherin by immuno-staining in control and treated cells (Figure 4a). Whereas almost no changes in E-cadherin expression was observed in HCT-116 and SW480 cells, the strong E-cadherin staining in SW620 cells clearly indicated that cell-cell contacts were re-established following treatment with PDGF and Y27632. Quantification of adherens junctions in SW620 cells confirmed the synergistic effect of PDGF and Y27632.

### Combined restoration of Cdc42 and Rac1 activity and inhibition of ROCK impair SW620 invasive properties

**Figure 5:**
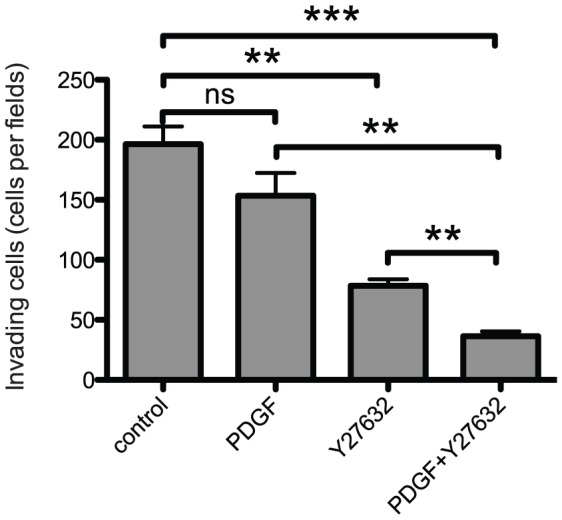
PDGF and Y27632 impair cancer cell invasion. The invasiveness of SW620 colon cancer cells following treatment or not (control) with PDGF and/or Y27632 was quantified by using Matrigel invasion assays as described in Materials and Methods. Values are the mean +/− SD (error bars) of at least three independent experiments and the statistical significance was calculated using the unpaired t-test. * p<0.05, ** p<0.01, *** p<0.001, ns non-significant.

As combined PDGF and Y27632 treatment led to the re-establishment of adherens junctions in the invasive SW620 cell line, we then tested directly the effect of PDGF and Y27632 treatment on the invasiveness of these cells in Matrigel invasion assays. Once again, the association of PDGF and Y27632 was more effective than the single treatments in inhibiting the invasive properties of SW620 cells (Figure 5). PDGF and Y27632 had almost no effects on HCT-116 and SW480 invasiveness (data not shown). Altogether, our data suggest that the invasive behaviour of the metastatic colon cancer cell line SW620 can be dramatically impaired by the combined use of PDGF and Y27632 that leads to amoeboid to epithelial transition and re-establishment of E-cadherin-dependent adherens junctions.

## Discussion

In this study, we investigated the possible relationship between invasiveness and activity level of the main Rho GTPases in a collection of colorectal cancer cell lines. We show that the ability to invade of these colorectal cancer cell lines is correlated with a marked decrease in Cdc42 and Rac1 activity and an increase in ROCK, but not RhoA, activity. As this molecular phenotype was associated with a round, blebbing cell morphology we then asked whether the normal epithelial morphology could be restored by inhibiting ROCK with Y27632 and reactivating Cdc42 and Rac1 with PDGF. Indeed, in the invasive SW620 cell line, the combined treatment with PDGF and Y27632 arrested blebbing and cells acquired a flattened and more spread morphology. Under these conditions, adherens junctions were also restored as indicated by the re-appearance of E-cadherin expression at cell-cell contacts. This morphological conversion was associated with a strong reduction in their ability to invade through Matrigel. It is highly conceivable that these features (cell-cell contacts/morphology and invasion) are directly linked.

Our data indicate that a strong decrease in Cdc42 and Rac1 activity is clearly associated with increased colon cancer invasiveness. These findings corroborate those obtained in other epithelial cell lines. In Madin Darby canine kidney (MDCK) epithelial cells, activation of Cdc42 and Rac1 has been implicated in the formation of adherens junctions [37]. Furthermore, the constitutively activated forms of Cdc42 and Rac1 can prevent Hepatocyte Growth Factor (HGF)-induced cell scattering by increasing E-cadherin-mediated cell-cell adhesion [37]. Our results extend these observations to human colon carcinoma cells. Specifically, upon restoration of the levels of GTP-bound Cdc42 and Rac1 by PDGF treatment, SW620 cell blebbing activity was strongly abolished and cells spread with the formation of filopodia and lamellae. In addition, we could confirm that expression of constitutively active Cdc42 or Rac1 inhibits SW620 invasive properties (data not shown). Moreover, PDGF also favoured the E-cadherin- dependent re-establishment of adherens junctions. Other studies have shown that activation of Rac1 results in E-cadherin-mediated cell-cell adhesion and, subsequently, inhibition of migration and invasion of epithelial cells [38] [39] [40] [41]. On the contrary, in hepatic tumours, PDGF is involved in the maintenance of EMT [42]. Moreover, Cdc42 and Rac1 might also play a key role in cell migration and invasion through the formation of filopodia and lamellipodia. Thereby, control of cell-cell contact to maintain cells as a cohesive epithelium and regulation of cell migration to favours invasion could be considered as two antagonistic effects of Cdc42 and Rac1 on tumour progression. In our study, PDGF treatment restored E-cadherin-dependent cell-cell contacts, but it was not sufficient to prevent invasion when used alone. Indeed, PDGF needed to be combined with ROCK inhibition to display a strong, additive effect on invasiveness. Our results thus indicate that each pathway displays independent features and that inhibition of invasion might be envisaged through effective manipulation of both pathways. However, these findings are associated with the amoeboid mode of migration of the SW620 cell line used in this study. As cancer cells can invade using different modes of migration, we do not know whether this modulatory strategy could be applied to other types of cancer cell invasion.

ROCK also plays a central role in the RhoA-dependent inhibition of Rac1 [22], [23], [43], [44], [45]. Recent advances have identified FilGAP, a filamin-binding protein endowed with a Rac1-specfic Rho GTPase-activating function, as a crucial mediator of ROCK-induced inhibition of Rac1. Following phosphorylation by ROCK, the RacGAP activity of FilGAP is stimulated and, as a consequence, FilGAP induces bleb formation and suppresses cell spreading and leading-edge formation, which are characteristics of Rac1 activity inhibition [22]. Moreover, ROCK signalling also activates ARHGAP22 (another RacGAP) that, in turn, inhibits Rac1 activation to avoid suppression of the blebbing-dependent (amoeboid?) movement [23]. However, RhoA can also mediate Rac1 activation and subsequent lamellipodia formation through recruitment of mDia, which is associated with membrane ruffles [43], [46]. Considering this double role, the absence of correlation between RhoA activation and cell invasion we report here is not surprising. ROCK activity is more likely to be associated with invasiveness, whereas the influence of RhoA oscillates between mDia and ROCK activation, depending on the cell type. Accordingly, the effects of the C3 exoenzyme, a Rho inhibitor, and of Y27632, a ROCK inhibitor, are very different. In LPA-stimulated Swiss 3T3 cells, Y27632, but not C3, treatment increased Rac1 activity and induced membrane ruffle formation [43].

Finally, one of the main findings in our study is that ROCK, but not RhoA, activity is related to invasiveness in our collection of colon cancer cell lines. This raises the question of the signalling pathway(s) that selectively activates ROCK in the more invasive cell lines. We can speculate that other ROCK activators might relieve RhoA activity in its absence. For instance, RhoC, which mainly promotes invasiveness [47], [48], [49], [50], [51], stimulates and interacts with ROCK more strongly than RhoA [52]. In agreement with this, RhoC expression and activation coincide with EMT of colorectal cancer cells that is associated with increased aggressiveness during tumorigenesis [53]. Taken together these data suggest that Rho GTPases may function in a compensatory, yet distinct, manner [54]. However, our knowledge on the involvement of Rho GTPases in cancer invasion is heavily dependent on the over-expression of constitutively activated forms. This might disturb the natural balance of Rho GTPase regulators and effectors. For instance, exposure of cells to high levels of active RhoA should favour signalling through ROCK, whereas low levels of RhoA promote signalling through mDia [52]. Therefore, the over-expression of a constitutively activated form of RhoA will promote ROCK-dependent invasiveness and might obscure the mDia antagonistic pathway. Moreover, accumulating evidence indicates that the GTPase signalling outcome depends not only on the effectors, but also on the GEFs that will drive the GTPases and the effectors in close proximity, creating micro-areas with high local concentrations of effectors [55] [56]. As a consequence, RhoA activity cannot totally account for the ROCK activation. Moreover, the crude RhoA activity might also be not fully indicative of which signalling pathway (i.e., GEFs and associated effectors) is engaged.

In conclusion, our work provides important insights on how Rho GTPases activation impacts on colorectal tumour progression and more precisely on the invasiveness of blebbing colorectal cells. Our central finding is the interplay between ROCK and Cdc42 and Rac1 activities. These signalling pathways have opposite effects on invasion and their fluctuations are partly mutually related, although our data also suggest the existence of independent intrinsic contributions. This would have important consequences on the strategies for the development of new anticancer drugs. Focusing only on the overall elevated expression levels of Rho GTPases in cancer might lead to therapeutic drawbacks if their opposing effects are not taken into account. Indeed, designing highly selective Rho GTPase signalling modulators and combining their effects might offer greater therapeutic opportunities.

## Materials and Methods

### Cell culture and reagents

The normal colon cell lines FHC, CoN and the colorectal cancer cell lines HCT116, CaCO2, LS174T, SW480, HT29, WidR, LoVo, SKCo1, SW620 and CoLo205 were purchased from ATCC and cultured as recommended. Cells were treated with 40 ng/ml PDGF-BB (Upstate) and 10 µM Y27632 (Calbiochem). Protein lysates were obtained by brief sonication of treated cells in 2× Laemmli buffer. Then, protein samples were resolved on 12% SDS-PAGE gels, immunoblotted and analysed with antibodies against phosphorylated Cofilin (on Ser3 from Santa Cruz Biotechnology, Inc.), total Cofilin (Cytoskeleton), phosphorylated MLC2 (T18/S19 from Cell Signaling), total MLC2 (Sigma-Aldrich) or alpha-Tubulin (Sigma-Aldrich). For evaluating the percentage of elongated cells, we used a published protocol [23]. Specifically, a cell was considered elongated when its longest dimension was twice the shortest one and when it showed at least one protrusion. For E-cadherin junction quantification, we scored as positive every cell with at least one E-cadherin junction with one or more neighbouring cells.

### Invasion assay

Quantification of cell invasion was performed in Transwell cell culture plates containing fluorescence-blocking polycarbonate porous membrane inserts (pore size of 8 µm; Fluoroblock; BD Biosciences). 100 µl Matrigel with reduced growth factors (a commercially prepared reconstituted basement membrane from Englebreth-Holm-Swarm tumours; BD Biosciences) was put in the upper chamber of the Transwell dish. Cells in monolayer were treated or not (controls) with PDGF and Y27632 for 2 h before trypsinization and then were plated (5.10^4^) in serum-free medium on top of the thick layer (around 500 µm) of Matrigel. The upper and lower chambers were then filled with serum-free DMEM and DMEM with 10% FCS, respectively, thus establishing a soluble gradient of chemo-attractant to promote cell invasion through Matrigel. Inhibitors were added immediately after cell plating at the aforementioned concentrations. Cells were allowed to invade at 37°C and 5% CO_2_ through Matrigel before being fixed in 3.7% formaldehyde for 15 minutes. Nuclei were stained with Hoechst 33342 (Sigma). Cells that had invaded through Matrigel were detected on the lower side of the filter by fluorescence and counted. Six fields per filter were counted and each assay was performed at least three times in triplicate for each cell line.

### GTPase activity assay

The GTPase activity assays were performed as described [57]. Briefly, 3.10^6^ cells were lysed before incubation with the GST–PAK fusion protein (PAK = the Cdc42 binding domain of human PAK1B, amino acids 56–272), for Cdc42 and Rac1 activity, or with the GST-RBD fusion protein (RBD = the Rho-binding domain of human Rhotekin, amino acids 7–89), for RhoA, coupled to Glutathione–Sepharose beads (Cytoskeleton). After precipitation, complexes were washed four times with lysis buffer, eluted in SDS–PAGE sample buffer, immunoblotted and analysed with antibodies against Cdc42, Rac1 (BD Transduction Laboratories) and RhoA (Santa Cruz Biotechnology, Inc.). Aliquots taken from supernatants prior to precipitation were used to quantify the total GTPases present in the cell lysates.

### Immunofluorescence

Cells seeded on coverslips were treated when about 50% confluent and then fixed in 3.7% formalin in phosphate-buffered saline (PBS) for 5 minutes followed by a 15 minutes permeabilization with 0.1% Triton-X100 (in PBS) and incubation in PBS containing 0.1% bovine serum albumin (BSA). Expression of E-cadherin was visualized after a 60 minutes incubation with the anti-E-cadherin monoclonal antibody HECD1 (ZYMED laboratories) (1∶200 dilution in PBS/BSA), followed by incubation with an affinity-purified fluorescein-conjugated goat anti-mouse antibody (Cappel-ICN) (1∶40 dilution). Cells were stained simultaneously for F-actin using 0.5 U/ml rhodamine-conjugated phalloidin (Sigma). Cells were washed in PBS, mounted in Mowiol (Aldrich) and observed using a DMR B microscope (Leica, Germany) with a PL APO 40× objective (NA 1.0, Zeiss, Germany). Slides were illuminated by a 100 W HBO 103W/2 light bulb (Osram, Germany). Images were captured with an ORCA 100 (B/W) 10 bits cooled CCD camera, a C 4742-95 controller and the HIPIC controller program run by a PC-compatible microcomputer (Hamamatsu, Japan). Images were saved in the TIFF format for processing and mounting with Microsoft Illustrator.

### Time-lapse imaging

Time-lapse differential interference contrast microscopy was performed using a Leica DMIRE2 inverted microscope with an automatic shutter, sample heater (37°C) and CO_2_ incubation chamber. Images were captured with the micromax CCD camera (1300Y/HS) imaging software, converted to TIFF files and were edited and compiled with Metamorph into AVI movies.

## Supporting Information

Video S1 **DIC time-lapse video of untreated HCT116 cells.** Pictures were captured every 3 seconds for 20 minutes.
(AVI)

Video S2 **DIC time-lapse video of HCT116 treated with PDGF and Y27632 for 24 hours.** After treatment, pictures were captured every 3 seconds for 20 minutes.
(AVI)

Video S3 **DIC time-lapse video of untreated SW480 cells.** Pictures were captured every 3 seconds for 20 minutes.
(AVI)

Video S4 **DIC time-lapse video of SW480 cells treated with PDGF and Y27632.** After 24 hours of treatment, pictures were captured every 3 seconds for 20 minutes.
(AVI)

Video S5 **DIC time-lapse video of untreated SW620 cells.** Pictures were captured every 3 seconds for 20 minutes.
(AVI)

Video S6 **DIC time-lapse video of SW620 cells treated with PDGF and Y27632.** After 24 hours of treatment, pictures were captured every 3 seconds for 20 minutes.
(AVI)

Video S7 **DIC time-lapse video of SW620 cells treated with PDGF and Y27632.** Pictures were captured every 3 seconds for 20 minutes before treatment and every 15 minutes for 18 hours after adding the treatment.
(AVI)
